# Seasonal Distribution, Virus-Specific Hematologic Profiles, and Predictors of Hospitalization in Children with Respiratory Viral Infections

**DOI:** 10.3390/children13081040

**Published:** 2026-08-04

**Authors:** Eda Özaydın, Seher Açar Bilge, Tuğçe Özbilgiç Demiröz, Handan Akkuş Karabacak, Elif Benderlioğlu, Aysun Yahsi

**Affiliations:** 1 Department of General Pediatrics, Ankara Bilkent City Hospital, University of Health Sciences, Ankara 06800, Türkiye; 2 Department of General Pediatrics, Ankara Bilkent City Hospital, Ministry of Health, Ankara 06800, Türkiyeelifbenderlioglu@gmail.com (E.B.); 3 Department of Pediatric Neurology, Gulhane Research and Training Hospital, University of Health Sciences, Ankara 06010, Türkiye; 4 Department of Pediatric Infectious Diseases, Ankara Bilkent City Hospital, Ministry of Health, Ankara 06800, Türkiye

**Keywords:** respiratory viral infections, multiplex PCR, seasonal distribution, hematological parameters, children

## Abstract

**Highlights:**

**What are the main findings?**

- Distinct virus-specific hematologic profiles were identified, with the most pronounced alterations observed in adenovirus and influenza infections.
- Respiratory viruses exhibited characteristic seasonal distribution and coinfection patterns, while younger age and elevated CRP were independent predictors of hospitalization.

**What are the implications of the main findings?**

- Recognition of virus-specific hematologic profiles, together with seasonal circulation and coinfection patterns, may improve the clinical evaluation of children with respiratory viral infections.
- Early assessment of age and CRP may support risk stratification and timely management of children at increased risk of hospitalization.

**Abstract:**

**Background**: Respiratory viral infections are a major cause of morbidity in children worldwide; however, several aspects remain incompletely understood. In particular, hematological changes associated with specific viral pathogens are not well defined, and their potential diagnostic value remains uncertain. In addition, the seasonal distribution of respiratory viruses may vary between years and geographical regions **Methods**: This retrospective study analyzed pediatric patients presenting to the outpatient clinics at Ankara Bilkent City Hospital between September 2024 and May 2025 who underwent multiplex PCR testing for respiratory viruses. Demographic characteristics, viral etiology, hematological parameters and hospitalization status were analyzed. **Results**: A total of 1143 patients tested positive for at least one viral pathogen; 91.1% had monoinfection and 8.9% had coinfection. The most commonly detected pathogens were rhinovirus/enterovirus, influenza A and respiratory syncytial virus, with frequencies of 23.7%, 17.7% and 13.7% respectively. Viral positivity peaked during winter, with distinct seasonal distribution patterns across pathogens. Coinfection and comorbid conditions were not significantly associated with hospital admission. In multivariable analysis, younger age and elevated CRP levels were identified as independent predictors of hospitalization. Significant differences in hematological parameters were observed among respiratory viruses, particularly in adenovirus and influenza infections. **Conclusions**: Respiratory viruses may exhibit distinct seasonal and hematological patterns. Younger age and elevated CRP levels are associated with an increased risk of hospitalization. Routine hematological parameters may provide additional value in clinical evaluation. Further studies are needed to validate these findings.

## 1. Introduction

Acute respiratory infections (ARIs) are common respiratory diseases in childhood and remain a leading cause of morbidity, hospitalization, and mortality worldwide [1,2]. Respiratory viruses continuously evolve through changes in their molecular characteristics, and pathogens such as respiratory syncytial virus (RSV), influenza A virus (IAV), influenza B virus (IBV), rhinovirus/enterovirus (RV/EV), adenovirus (AdV), human parainfluenza virus (HPIV), human coronaviruses (HCoVs), human metapneumovirus (HMPV) account for the majority of seasonal respiratory infections in children [3]. In the northern hemisphere, these viruses exhibit characteristic seasonal patterns: influenza viruses and RSV peak during winter, rhinoviruses are more prevalent in spring and autumn, and HPIV types 1 and 3 circulate predominantly in winter and spring–summer, respectively, whereas HMPV, AdV and human bocavirus (HBoV) may circulate throughout the year [4,5]. The epidemiology of respiratory viral infections in children has changed substantially in the post-pandemic period. During the COVID-19 pandemic, the widespread implementation of non-pharmaceutical interventions, including masking, social distancing, school closures, and improved hand hygiene, markedly reduced the circulation of most respiratory viruses. Following the relaxation of these measures, however, several studies reported atypical seasonal patterns characterized by off-season outbreaks, altered timing of epidemics, and shifts in the predominance of circulating viruses, particularly RSV and influenza viruses. In contrast, RV and AdV circulation was less affected and resumed rapidly after restrictions were lifted [6,7]. These epidemiological changes have varied across geographical regions and seasons, underscoring the importance of continuous local surveillance to monitor evolving viral circulation and to support clinical diagnosis and public health planning in children.

Previous studies have investigated risk factors associated with hospitalization in respiratory tract infections. In a meta-analysis including 30 studies, several factors were identified as significant predictors of RSV-related acute lower respiratory infection (ALRI), including chronic diseases, younger age, viral coinfections and undernutrition. For influenza-related ALRI, chronic underlying conditions and age between 6 and 24 months were identified as important risk factors for poor outcomes. In SARS-CoV-2 (severe acute respiratory syndrome coronavirus 2)-associated ALRI, cardiovascular disease, immunosuppression, chronic kidney disease, diabetes and hypertension have been reported as major risk factors for mortality [8]. However, despite these findings, the evidence remains heterogeneous and insufficient to fully explain risk stratification across different populations.

Viral coinfections occur in approximately 10–20% of respiratory viral infections [9]. However, their clinical impact remains controversial, as some studies report an association with disease severity, whereas others find no significant effect on clinical outcomes [10,11,12]. Further research is needed to clarify their clinical relevance.

Hematologic and inflammatory parameters may reflect the host immune response during viral infections. Huang et al. reported significant differences in routine blood parameters among patients with COVID-19, IAV, and RSV early in the disease course suggesting their potential utility in differentiating these infections [13]. In addition, numerous studies in COVID-19 patients have demonstrated correlations between hematological parameters and disease severity [14,15,16]. Following the COVID-19 pandemic, several respiratory viruses, particularly RSV, have re-emerged with altered seasonality and, in some populations, increased disease severity. COVID-19’s effect was not limited to respiratory infectious diseases, but it affected other diseases, including urinary tract and gastrointestinal infections or menengitis. These epidemiological changes have been attributed to reduced population immunity after prolonged periods of limited viral exposure (“immunity gap” or “immune debt”) [17]. These changes may also influence host inflammatory and hematological responses. However, direct comparisons of virus-specific hematological profiles between the pre- and post-pandemic periods remain limited. Therefore, our study provides important data describing hematological responses to respiratory viral infections in children during the post-pandemic era.

The aim of this study was to evaluate virus-specific hematological parameters and predictors of hospitalization in children, and to characterize the seasonal distribution and coinfection patterns of common respiratory viruses in a large pediatric cohort.

## 2. Methods

### 2.1. Study Design, Population and Data Collection

This retrospective observational study was conducted at Ankara Bilkent City Hospital between September 2024 and May 2025. Medical records of pediatric patients who presented with symptoms of respiratory tract infection and underwent respiratory viral panel testing were reviewed. Age, gender, date and season of presentation, history of comorbid conditions (including chronic pulmonary disease, congenital heart disease, asthma/recurrent wheezing, neurologic disorders, and metabolic diseases), and laboratory parameters were retrospectively collected from the electronic medical records.

Children aged 1–72 months with a clinical diagnosis of upper or lower respiratory tract infection and an available respiratory multiplex PCR result were eligible for inclusion. Patients with underlying chronic hematologic or oncologic diseases, immunodeficiency, receipt of systemic corticosteroid or immunosuppressive therapy, or incomplete clinical or laboratory data were excluded. Other comorbid conditions were not considered exclusion criteria and were recorded as baseline clinical characteristics.

### 2.2. Laboratory Parameters

Laboratory data obtained at presentation included hemoglobin (Hb), white blood cell count (WBC), absolute neutrophil and lymphocyte counts (ANC, ALC), platelet count (Plt) and mean platelet volume (MPV), absolute eosinophil count (AEC), absolute monocyte count (AMC), neutrophil–lymphocyte ratio (NLR), delta neutrophil index (DNI), and C-reactive protein (CRP).

Nasopharyngeal swab samples were collected using standard procedures and transported to the microbiology laboratory. Viral pathogens were detected using a multiplex real-time PCR assay (Rotor-Gene Q, QIAGEN, Germantown, MD, USA). The panel included AdV, HBoV, HCoV, RV/EV, IAV, IBV, HPIV, RSV A/B, HMPV, Mycoplasma pneumoniae and SARS-CoV-2.

A single detected agent was classified as monoinfection, whereas detection of ≥2 viruses was classified as coinfection.

### 2.3. Seasonal Classification

Seasons were categorized as follows: autumn (September–November), winter (December–February), and spring (March–May).

### 2.4. Outcomes

Seasonal patterns of respiratory viral infections, independent predictors of hospitalization and virus-specific hematological profiles.

### 2.5. Statistical Analysis

The data obtained in this study were analyzed using the IBM SPSS Statistics software, version 23 (SPSS Inc., Armonk, New York, NY, USA, IBM Corp., USA). Since the sample size was greater than 30, normal distribution of the samples was assumed according to the Central Limit Theorem.

Descriptive statistics were presented as mean and standard deviation for continuous variables, and as frequency and percentage for categorical variables. For comparisons between groups, the Independent Samples *t*-Test was used for continuous variables that showed a normal distribution. The Pearson Chi-Square (χ^2^) test and Fisher’s Exact Test were applied for the comparison of categorical variables. For variables found to be significant in the Pearson Chi-Square analysis, pairwise comparisons were conducted using the post hoc Bonferroni test.

To determine the factors affecting hospitalization, both univariable and multivariable binary logistic regression analyses were performed. The model’s goodness of fit was evaluated using the Omnibus Tests of Model Coefficients, its explanatory power was assessed with Cox & Snell R^2^ and Nagelkerke R^2^, and its classification performance was evaluated with the Classification Table (overall accuracy rate). Relationships between variables were examined using a correlation matrix, and no multicollinearity was detected. For all analyses, a *p*-value < 0.050 was considered statistically significant.

### 2.6. Ethics

The study was consistent with the principles of the Declaration of Helsinki and was approved by the Ethics Committee of Ankara Bilkent City Hospital (Date: 16 April 2025, reference number: TABED 2-25-1083).

## 3. Results

### General Characteristics of the Patients

This study included 1143 patients who tested positive for respiratory viruses. A single pathogen was detected in 1041 patients, dual pathogens in 96 patients, and three pathogens in six patients. There were 496 (43.4%) female and 647 (56.6%) male patients. The highest positivity rate was observed during the winter season (49.8%) and was subsequently detected in autumn and spring (29.8% and 20.4%, respectively). Underlying comorbid conditions were present in 71 patients (6.2%), including chronic pulmonary disease, congenital heart disease, asthma/recurrent wheezing, neurologic disorders, and metabolic diseases.

The most common pathogens were RV/EV (23.7%), followed by IAV (17.7%) and RSV A/B (13.7%) (Table 1). Other frequently identified agents included AdV (10.9%), seasonal coronaviruses (7.0%), and HPIV (6.0%). Among the 1143 patients evaluated, single viral infections constituted the majority (91.1%), whereas multiple infections were relatively uncommon (8.9%). Among coinfected patients, RV/EV was the most frequently detected pathogen, identified in 43 cases. The highest proportions of coinfection were observed in HBoV and AdV infections, whereas the lowest proportions were found in IAV, IBV, and SARS-CoV-2 infections.

Patients who required hospitalization were significantly younger than outpatients (25.08 ± 21.81 vs. 32.02 ± 22.05 months, *p* = 0.009) (Table 2). Hospitalized patients also had significantly lower hemoglobin levels (*p* = 0.008) and higher WBCs (*p* = 0.010). Although ANC, AMC and DNI tended to be higher in the hospitalized group, these differences did not reach statistical significance. Notably, CRP levels were significantly elevated in hospitalized patients (33.83 ± 66.27 vs. 14.96 ± 29.55 mg/L, *p* = 0.019).

Hospitalization rates were significantly higher among RSV-positive patients (26.7% vs. 14.1%) and lower among IAV-positive patients (6.7%) (*p* = 0.002). No statistically significant association was found between coinfections and hospitalization (*p* > 0.05).

**Figure 1:**
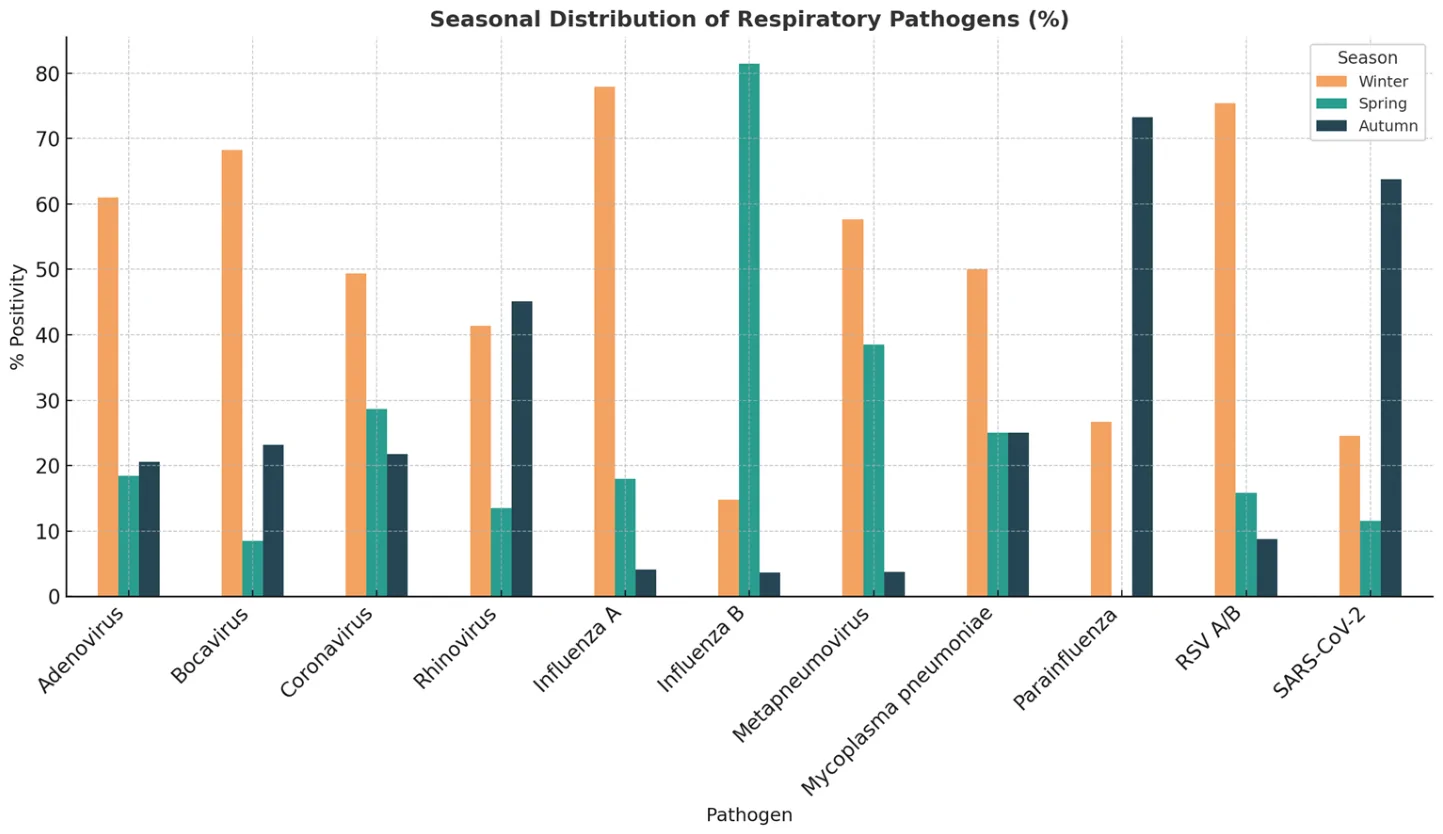
Seasonal distribution of respiratory pathogens (%).

Figure 1 shows the percentage distribution of respiratory pathogens across winter, spring, and autumn seasons. RSV A/B, IAV, AdV, HBoV and HMPV demonstrated clear winter predominance. RV/EV and HPIV were most commonly detected in autumn, whereas IBV showed a peak in spring. SARS-CoV-2 positivity was highest in autumn and lowest in spring. These trends illustrate distinct seasonal circulation patterns among viral pathogens in early childhood.

Respiratory pathogens demonstrated significant seasonal variability (Figure 1). AdV and HBoV infections were significantly more common during winter, with positivity rates of 61% and 68.3%, respectively. In both pathogens, detection rates decreased markedly in spring (*p* = 0.018 and *p* = 0.002, respectively). IAV infections peaked in winter (77.9%), while RSV A/B positivity was highest in winter (75.4%). IBV infections showed a distinct seasonal peak in spring (81.5%) (*p* < 0.001). HMPV infections were predominantly detected in winter and spring, whereas only a small proportion of cases occurred in autumn (3.8%). RV/EV and HPIV were most frequently detected in autumn, with positivity rates of 45.1% and 73.3%, respectively. HPIV infections were not detected during spring (*p* < 0.001). SARS-CoV-2 infections were also significantly more common in autumn (63.8%) compared with other seasons (*p* < 0.001). *M. pneumoniae* infections were distributed relatively throughout the year, and no significant seasonal variation was observed (*p* = 0.741).

Univariable logistic regression analysis revealed that age, WBC, AMC, DNI, RSV A/B and RV/EV positivity are associated with hospitalization (Table 3). In univariable logistic regression, RV/EV positivity was associated with 1.7-fold higher odds of hospitalization, while RSV A/B positivity was associated with approximately 2.2-fold higher odds. In contrast, IAV positivity was associated with significantly lower odds of hospitalization (*p* = 0.007).

A multivariable logistic regression analysis identified younger age and higher CRP levels as independent predictors of hospital admission (Table 4). Younger age was independently associated with hospitalization (B = –0.018, *p* = 0.011, OR = 0.982, 95% CI: 0.968–0.996), indicating that each additional month of age decreased the odds of hospitalization by approximately 1.8%. Higher CRP levels were positively associated with hospital admission (B = 0.007, *p* = 0.015, OR = 1.007, 95% CI: 1.001–1.014), suggesting that each 1 mg/L increase in CRP increased the odds of hospitalization by about 0.7%.

Across the evaluated viral pathogens, distinct hematologic and inflammatory profiles were observed.

Table 5: AdV-positive patients were characterized by significantly increased WBC, ANC, ANC %, CRP and NLR, whereas eosinophil levels were reduced. HBoV infections were associated with younger age and higher eosinophil counts, without significant changes in other laboratory parameters. RV/EV-positive children exhibited significantly higher WBC, ANC, ALC, AEC and platelet counts compared with non-RV/EV patients.

In contrast, IAV infection was associated with significantly lower WBC, ANC, ALC, AMC, AEC and platelet counts, accompanied by higher NLR and older age. Similarly, IBV infection was characterized by lower WBC, ANC, ALC, and AEC levels, as well as decreased DNI, platelet counts, and CRP levels, together with increased MPV and older age. The highest mean age was observed among patients with IAV and IBV infections. HPIV infection was associated with younger age and significantly lower leukocyte, neutrophil, eosinophil, and NLR values. RSV-positive patients also demonstrated younger age, higher lymphocyte counts, lower NLR values, and reduced CRP levels.

SARS-CoV-2-positive patients showed significantly lower leukocyte and neutrophil parameters, reduced NLR and CRP levels, and younger age. Although monocyte counts were higher than those in the non-SARS-CoV-2 group, the difference was not statistically significant.

Patients with *M. pneumoniae* infection were significantly older than those without the infection; however, no significant differences in hematologic parameters were observed.

Comparison of respiratory virus positivity and laboratory findings between the two age groups revealed several age-related differences (Table 6). Rhinovirus/enterovirus (29.9% vs. 19.5%, *p* < 0.001), RSV A/B (17.1% vs. 11.5%, *p* = 0.010), and SARS-CoV-2 (7.8% vs. 3.2%, *p* = 0.001) were detected more frequently in children aged 1–36 months, whereas IVA (14.3% vs. 27.8%, *p* < 0.001), IVB (2.5% vs. 8.3%, *p* < 0.001), and *M. pneumoniae* (2.0% vs. 4.1%, *p* = 0.041) were more common in those aged 37–72 months. No significant age-related differences were observed in the positivity rates of AdV, HBoV, HMPV, or HPIV. Children aged 1–36 months had significantly higher WBC, ALC, AMC, AEC and platelet counts than those aged 37–72 months (all *p* < 0.001). In contrast, children aged 37–72 months had significantly higher ANC (*p* = 0.006), neutrophil percentage (*p* < 0.001), NLR (*p* < 0.001), and CRP levels (*p* = 0.029). DNI and MPV did not differ significantly between the two age groups.

## 4. Discussion

In this study, we investigated virus-specific variations in hematological parameters, age-related differences in respiratory pathogen distribution, independent predictors of hospitalization, and the seasonal distribution and coinfection patterns of common respiratory viruses. The most commonly identified pathogens were RV/EV, IAV, and RSV. RV/EV was the most frequently detected pathogen in coinfections, whereas AdV and HBoV showed the highest coinfection rates. Respiratory viruses demonstrated significant seasonal variability, peaking during the winter months. Age-stratified analysis showed that RV/EV, RSV, and SARS-CoV-2 infections were more common in younger children, whereas influenza viruses and M. pneumoniae predominated in older children. In univariable analyses, WBC, AMC, DNI, RSV positivity, and RV/EV positivity were significantly associated with hospital admission. Neither coinfections nor comorbid conditions were associated with hospitalization. Among a broad range of clinical and laboratory parameters, younger age and elevated CRP levels emerged as independent predictors of hospitalization. Distinct virus-specific hematological profiles were observed, with the most pronounced alterations occurring in adenovirus and influenza infections.

Numerous studies have investigated risk factors associated with hospitalization in general pediatric wards and pediatric intensive care units (PICUs). Prematurity, younger age and comorbid conditions have been identified as factors associated with an increased risk of hospital admission [18,19]. In our study, younger age and RSV positivity were associated with increased disease severity consistent with previous reports [20,21,22]. Influenza infections have also been reported to be associated with younger age and the presence of comorbidities [23]; however, in our cohort, influenza was associated with a lower risk of severe disease. The widespread use of antiviral therapy in our center and the inclusion of patients presenting to outpatient clinics may have contributed to reduced hospitalization rates. Moreover, the high proportion of otherwise healthy children without chronic conditions may have further caused the lower hospitalization rate. In contrast, RSV infection showed a stronger association with severe disease, highlighting the importance of effective RSV prevention strategies including maternal RSV vaccination during pregnancy or long-acting monoclonal antibodies such as nirsevimab or clesrovimab for infants entering their first RSV season.

Viral coinfections are frequently observed in children; however, whether specific viral interactions enhance or diminish the severity of respiratory disease remains controversial. In a meta-analysis by Goka, the evidence regarding the impact of coinfections on disease severity was inconclusive [10]. Similarly, in our study, no significant differences were observed between single infections and coinfections in terms of hospitalization rates. The frequency of coinfections was lower than that reported in the literature. This finding may be attributed to patient characteristics, as our cohort consisted of patients presenting to general pediatrics and infectious diseases clinics, with a relatively low hospitalization rate. This study demonstrated that SARS-CoV-2 and IAV were more commonly observed in monoinfections, consistent with previous data [24,25]. Maio et al. reported that HBoV exhibited the highest cocoinfectionate (87.8%), likely due to prolonged viral shedding, with viral DNA persisting for up to 3 months in outpatients and up to 1 year in hospitalized children after acute infection [26]. In our cohort, HBoV, AdV and RV/EV also demonstrated notable coinfection tendencies, in agreement with the literature. Additionally, the coinfection rate of *M. pneumoniae* was relatively high (28.1%). The study period coincided with the global resurgence of *M. pneumoniae* infections observed in 2024 following the COVID-19 pandemic. The increased circulation of *M. pneumoniae*, particularly among children, may have contributed to the high detection rate of *M. pneumoniae* and the frequent occurrence of viral coinfections observed in this study [27,28].

The rate of viral positivity varied significantly across seasons, with the highest rate observed in winter (49.8%). While this finding is generally consistent with the known seasonality of respiratory viruses, some differences compared with previous studies were noted. Zhu et al. [1] reported that AdV infections peaked in spring, whereas IAV and IBV in winter, and RSV in autumn. Zhao et al. found that IAV and IBV epidemics occurred predominantly in winter and spring, whereas AdV did not exhibit a distinct seasonal pattern and RSV peaked in winter [29]. Adenovirus infections occurred more frequently during winter in our cohort. Fang et al. also demonstrated a winter peak of AdV infections. These differences may reflect variations in geographical settings, population characteristics, and viral transmission patterns.

The COVID-19 pandemic has significantly altered the epidemiology of respiratory viruses, particularly in terms of seasonal distribution and peak incidence. Several studies from Türkiye have investigated these changes during and after the pandemic. Rhinovirus was the most frequently seen virus in these studies (excluding COVID-19) [3,30] The frequency of parainfluenza virus infections was found to be increased during the summer months, representing a novel finding [30]. In our cohort, parainfluenza infections were most frequently observed in autumn; however, the lack of summer data limits direct comparison. In another study conducted at our center, influenza virus was detected in 30.1% and HBoV in 28.3% of 1465 hospitalized patients [31]. These differences may be explained by variations in study design and population characteristics, as our study included outpatients, excluded emergency department cases, and had a relatively low prevalence of comorbidities. Despite changes in the epidemiology of respiratory viruses after the COVID-19 pandemic, rhinovirus has remained the most frequently detected pathogen. SARS-CoV-2, on the other hand, appears to be evolving toward an endemic pattern with increasing evidence of seasonal circulation; however, longer-term surveillance is required to confirm this transition.

We compared hematological parameters between virus-specific groups, including AdV vs. non-AdV and RSV vs. non-RSV infections, to explore their potential role as supportive diagnostic markers in the absence of molecular testing. Hematological parameters—including WBC, neutrophil, lymphocyte, platelet, and eosinophil counts—varied significantly among respiratory viral pathogens, supporting the concept that each virus may induce a distinct immuno-hematological profile.

Adenovirus infection was associated with the most pronounced inflammatory response, characterized by elevated CRP, WBC, and neutrophil counts, along with low eosinophil levels. These findings are consistent with previous reports indicating that AdV infections may mimic bacterial infections in terms of clinical, laboratory, and radiological features [32]. The highest WBC and ANC were observed in AdV and RV/EV infections.

Eosinopenia is often considered a marker of infection, although there is currently no universally accepted cutoff value The clinical utility of eosinopenia has been explored in various settings, including early neonatal sepsis, differentiation between bacterial and aseptic meningitis, and early diagnosis of COVID-19 infection [33,34,35]. Although the precise role of eosinophils in viral infections remains unclear, current evidence suggests that peripheral blood eosinophil count (EC) may serve as a predictive and prognostic biomarker for disease outcomes [36,37,38]. In the current study, we found no significant association between eosinophil levels and hospital admission but we observed low eosinophil levels in AdV-, IAV-, IBV-, and HPIV-positive patients. As key components of the innate immune system, eosinophils exert protective effects through their anti-infective and anti-inflammatory activities [39]. However, they can also contribute to pathological conditions such as asthma and atopic dermatitis. In our cohort, higher eosinophil levels were observed in RV/EV and HBoV infections. These findings are noteworthy, as these viruses have been implicated in the development of asthma and allergic diseases.

Low lymphocyte levels were observed in IAV and IBV patients consistent with the literature [40]. In contrast, RSV and RV/EV infections showed a lymphocyte-predominant profile and SARS-CoV-2 infection was associated with lower neutrophil levels and higher monocyte counts. Monocytosis has previously been identified as a potential marker of innate immune activation in pediatric COVID-19 [41]. In our study, elevated monocyte levels were also observed in hospitalized patients, suggesting a potential association with disease severity.

Age-stratified analysis confirmed the expected physiological differences in hematological parameters between younger and older children. However, the observed virus-associated hematological alterations could not be explained by age alone. Several respiratory viruses exhibited distinct hematologic profiles that differed from the expected age-related patterns. For example, RV/EV infection was associated with higher neutrophil, lymphocyte, and eosinophil counts despite occurring predominantly in younger children. Likewise, AdV, influenza A/B, and HPIV infections were consistently associated with eosinopenia, suggesting that reduced eosinophil counts reflect a virus-specific host immune response rather than physiological age-related variation. In contrast, SARS-CoV-2 infection was characterized by neutropenia, whereas AdV exhibited a pronounced inflammatory profile irrespective of age distribution. Collectively, these findings suggest that virus-specific host immune responses, rather than age alone, are important determinants of the hematological alterations observed in children with respiratory viral infections.

This study has several limitations. First, its retrospective design may have introduced selection bias and limited the availability of some clinical data. Second, the study was conducted in a single center, which may limit the generalizability of the findings. Third, the study population consisted of children initially presenting to the outpatient clinic. As only a relatively small proportion required hospitalization, the low number of hospitalized patients and the limited prevalence of severe disease and comorbid conditions may have affected the identification of hospitalization-related risk factors. In addition, the absence of summer data limited the evaluation of seasonal patterns for certain respiratory viruses, because the summer months were outside the predefined study period and were therefore unavailable at the time of data extraction. Despite these limitations, the study has several strengths, including a relatively large overall cohort and the comprehensive evaluation of multiple respiratory viruses.

In conclusion, respiratory viral infections in children exhibit distinct seasonal and hematological patterns. Age-related differences were observed in pathogen distribution, with RV/EV, RSV A/B, and SARS-CoV-2 detected more frequently in younger children, whereas IAV, IVB, and *M. pneumoniae* predominated in older children. Distinct virus-specific hematological profiles, particularly in AdV and influenza infections, suggest that routine hematological parameters may provide valuable insights into host immune responses and aid the clinical evaluation of children with respiratory viral infections. Although several hematological parameters were associated with hospitalization in the univariable analysis, only younger age and elevated CRP remained independent predictors indicating that most virus-specific hematological alterations are more likely to reflect pathogen-specific immune responses than disease severity. Routine hematological parameters may complement clinical assessment, particularly in settings where molecular diagnostics are not readily available. Further prospective, multicenter studies are needed to validate these findings and to elucidate the underlying biological mechanisms.

## Figures and Tables

**Table 1:** The number and percentage of respiratory viral infections.

Virus	Single N (%)	Multiple N (%)	Total N (%)
AdV	97 (71.3%)	39 (28.7%)	136 (10.9%)
HBoV	51 (62.2%)	31 (37.8%)	82 (6.6%)
HCoV	75 (86.2%)	12 (13.8%)	87 (7.0%)
RV/EV	254 (85.5%)	43 (14.5%)	297 (23.7%)
IAV	202 (91.0%)	20 (9.0%)	222 (17.7%)
IBV	52 (96.3%)	2 (3.7%)	54 (4.3%)
HMPV	22 (84.6%)	4 (15.4%)	26 (2.1%)
*M. pneumoniae*	23 (71.9%)	9 (28.1%)	32 (2.6%)
HPIV	59 (78.7%)	16 (21.3%)	75 (6.0%)
RSV A/B	141 (82.5%)	30 (17.5%)	171 (13.7%)
SARS-CoV-2	65 (94.2%)	4 (5.8%)	69 (5.5%)
Overall	1041	210	1251

**Table 2:** Comparison of hematologic and inflammatory parameters between hospitalized and non-hospitalized patients.

Hospitalization State		Number of Patients	Mean	Std. Deviation	*p*
Age (month)	N	1068	32.02	22.05	0.009
	H	75	25.08	21.81	
Hb (g/dL)	N	477	12.12	1.21	0.008
	H	75	11.71	1.33	
WBC (/mm^3^)	N	477	8983.71	3805.09	0.010
	H	75	10,585.33	5032.50	
ANC (/mm^3^)	N	477	4582.52	3863.33	0.079
	H	75	5519.07	4295.14	
ANC %	N	477	47.13	18.87	0.800
	H	75	47.74	21.73	
ALC (/mm^3^)	N	477	3452.77	1933.75	0.392
	H	75	3661.47	2131.02	
AMC (/mm^3^)	N	476	609.12	372.70	0.090
	H	75	808.93	998.15	
AEC (/mm^3^)	N	476	158.45	190,53	0.328
	H	75	183.07	267.55	
DNI %	N	473	0.69	1.65	0.073
	H	75	1.20	2.32	
Plt (/mm^3^)	N	475	334,002	119,256	0.170
	H	75	357,733	140,699	
MPV (fL)	N	475	7.85	0.85	0.967
	H	75	7.84	1.02	
NLR	N	475	1.83	1.80	0.321
	H	75	2.10	2.29	
CRP (mg/L)	N	391	14.96	29.55	0.019
	H	73	33.83	66.27	

Abbreviation: Hemoglobin (Hb), white blood cell count (WBC), hematocrit (Hct), red blood cell count (RBC), mean corpuscular volume (MCV), absolute neutrophil count (ANC), absolute lymphocyte count (ALC), absolute monocyte count (AMC), absolute eosinophil count (AEC), platelet count, mean platelet volume (MPV), C-reactive protein (CRP), N: Non-hospitalized, H: Hospitalized.

**Table 3:** Univariable logistic regression analysis of factors associated with respiratory viral infections.

Variable	B	*p*	Exp(B)	%95 GA (Exp(B))
Age (month)	−0.016	0.009	0.985	0.973–0.996
Hb (g/dL)	−0.268	0.008	0.765	0.628–0.933
WBC (/mm^3^)	0.000	0.002	1.000	1.000–1.000
CRP (mg/L)	0.009	0.001	1.009	1.004–1.014
AMC (/mm^3^)	0.001	0.017	1.001	1.000–1.001
DNI (%)	0.125	0.026	1.133	1.015–1.265
RSV A/B	0.792	0.004	2.208	1.287–3.789
IAV	−1.273	0.007	0.280	0.112–0.703
RV/EV	0.508	0.043	1.662	1.017–2.717

**Table 4:** Multivariable logistic regression analysis of factors associated with respiratory viral infections.

Variable	B	*p*	Exp(B)	%95 GA (Exp(B))
Age (month)	−0.018	0.011	0.982	0.968–0.996
Hb (g/dL)	−0.049	0.664	0.952	0.763–1.189
WBC (/mm^3^)	0.000	0.257	1.000	1.000–1.000
CRP (mg/L)	0.007	0.015	1.007	1.001–1.014
AMC (/mm^3^)	0.000	0.307	1.000	1.000–1.001
DNI (%)	0.088	0.159	1.092	0.966–1.235
Constant	−1.340	0.314	0.262	—

**Table 5:** Comparison of hematologic and inflammatory parameters across viral pathogens.

Viral Etiology	Parameter	*N*	Mean ± SD	*p*
Adenovirus	Hb	73	11.72 ± 1.13	0.013
	WBC	73	11,161.23 ± 4516.42	<0.001
	ANC	73	6788.90 ± 3687.83	<0.001
	ANC (%)	73	57.89 ± 16.56	<0.001
	AEC	73	99.86 ± 118.87	<0.001
	NLR	73	2.62 ± 1.88	<0.001
	CRP	59	46.53 ± 59.96	<0.001
Human Bocavirus	Age (month)	82	29.51 ± 18.29	0.032
	AEC	44	320.23 ± 312.40	0.001
Rhinovirus/Enterovirus	Age (month)	297	27.19 ± 21.05	<0.001
	WBC	140	11,124.64 ± 4063.21	<0.001
	ANC	140	6139.64 ± 5734.07	<0.001
	ALC	140	4267.86 ± 2050.73	<0.001
	AEC	140	234.00 ± 234.81	<0.001
	Plt	140	388,128 ± 136,051	<0.001
Influenza A	Age (month)	222	40.24 ± 22.38	<0.001
	Hb	109	12.28 ± 1.07	0.037
	WBC	109	6905.14 ± 3074.69	<0.001
	ANC	109	3645.41 ± 2481.75	<0.001
	ANC (%)	109	50.51 ± 20.08	0.045
	ALC	109	2344.77 ± 1427.04	<0.001
	AMC	109	500.92 ± 278.57	0.002
	AEC	109	80.46 ± 137.79	<0.001
	Plt	109	286,853 ± 98,009	<0.001
	NLR	109	2.29 ± 2.21	0.020
	CRP	94	10.34 ± 14.09	<0.001
Influenza B	Age (month)	54	47.37 ± 22.07	<0.001
	HB	28	12.50 ± 0.82	0.008
	WBC	28	6130.00 ± 3106.28	<0.001
	ANC	28	3010.71 ± 2248.44	0.019
	ALC	28	2400.71 ± 1414.09	0.003
	AEC	28	74.29 ± 148.39	0.019
	DNI	28	0.35 ± 0.82	0.017
	Plt	28	221,214.29 ± 76,440.90	<0.001
	MPV	28	8.26 ± 1.09	0.010
	CRP	28	4.67 ± 7.13	<0.001
Human Parainfluenza Virus	Age (month)	75	26.57 ± 22.57	0.004
	WBC	36	7559.44 ± 3057.00	0.002
	ANC	36	3016.39 ± 2058.61	<0.001
	ANC (%)	36	39.18 ± 18.24	0.010
	AEC	36	88.89 ± 98.82	<0.001
	DNI	36	0.44 ± 0.89	0.048
	NLR	36	1.26 ± 1.59	0.047
RSV A/B	Age (month)	171	26.85 ± 19.18	<0.001
	ALC	74	3986.35 ± 2094.31	0.017
	NLR	73	1.39 ± 1.28	0.002
	CRP	64	10.36 ± 15.37	0.002
SARS-CoV-2	Age (month)	69	20.38 ± 21.20	<0.001
	WBC	30	7225.00 ± 2558.00	<0.001
	ANC	30	2433.00 ± 1660.83	<0.001
	ANC (%)	30	33.43 ± 18.72	<0.001
	DNI	30	0.38 ± 0.97	0.042
	NLR	30	0.99 ± 0.89	<0.001
	CRP	24	7.73 ± 10.29	<0.001

**Table 6:** Age-specific respiratory virus positivity and laboratory findings.

Variable	1–36 month	37–72 month	*p*
Adenovirus	81 (11.4)	55 (12.6)	0.573
Human Bocavirus	55 (7.8)	27 (6.2)	0.347
Human Coronavirus	48 (6.8)	39 (9)	0.206
Rhinovirus/Enterovirus	212 (29.9)	85 (19.5)	<0.001
Influenza A	101 (14.3)	121 (27.8)	<0.001
Influenza B	18 (2.5)	36 (8.3)	<0.001
Human Metapneumovirus	17 (2.4)	9 (2.1)	0.839
*M. pneumoniae*	14 (2.0)	18 (4.1)	0.041
Human Parainfluenza Virus	53 (7.5)	22 (5.1)	0.112
RSV A/B	121 (17.1)	50 (11.5)	0.010
SARS-CoV-2	55 (7.8)	14 (3.2)	0.001
WBC (/mm^3^)	9802.2 ± 4057.8	8365.3 ± 3832.5	<0.001
ANC (/mm^3^)	4315.5 ± 3736.6	5240.9 ± 4133	0.006
ANC (%)	39.91 ± 18.02	57.19 ± 16.18	<0.001
ALC (/mm^3^)	4286 ± 2036.2	2374.8 ± 1144.2	<0.001
AMC (/mm^3^)	707.8 ± 604.5	538.4 ± 309.5	<0.001
AEC (/mm^3^)	199.6 ± 225.4	109.7 ± 151.7	<0.001
DNI (%)	0.68 ± 1.76	0.86 ± 1.77	0.241
Plt (/mm^3^)	358,291.5 ± 121,117.2	307,784.5 ± 118,594.5	<0.001
MPV (fL)	7.87 ± 0.92	7.81 ± 0.80	0.410
NLR	1.27 ± 1.33	2.67 ± 2.18	<0.001
CRP (mg/L)	14.48 ± 33.97	22.61 ± 43.07	0.029

## Data Availability

The data presented in this study are available on request from the corresponding author. The data are not publicly available due to restrictions, e.g., privacy.

## Abbreviations

ARI	Acute respiratory infection
RSV	Respiratory syncytial virus
IAV	Influenza A virus
IBV	Influenza B virus
RV/EV	Rhinovirus/Enterovirus
AdV	Human adenovirus
HPIV	Human parainfluenza virus
hCoV	Human coronaviruses
HMPV	Human metapneumovirus
hBoV	Human bocavirus
ALRI	Acute lower respiratory infection
SARS-CoV-2	Severe acute respiratory syndrome coronavirus 2
Hb	Hemoglobin
WBC	White blood cell count
ANC	Absolute neutrophil count
ALC	Absolute lymphocyte count
AMC	Absolute monocyte count
AEC	Absolute eosinophil count
Plt	Platelet count
MPV	Mean platelet volume
CRP	C-reactive protein
PCR	Polymerase chain reaction
PICU	Pediatric intensive care unit
